# Sutureless Bioprostheses for Aortic Valve Replacement: An Updated Systematic Review with Long-Term Results

**DOI:** 10.3390/jcm13226829

**Published:** 2024-11-13

**Authors:** Giovanni Alfonso Chiariello, Michele Di Mauro, Emmanuel Villa, Marinos Koulouroudias, Piergiorgio Bruno, Andrea Mazza, Annalisa Pasquini, Serena D’Avino, Gaia De Angelis, Kiara Corigliano, Alberta Marcolini, Edoardo Zancanaro, Guglielmo Saitto, Paolo Meani, Massimo Massetti, Roberto Lorusso

**Affiliations:** 1 Department of Cardiovascular Sciences, Agostino Gemelli Foundation Polyclinic IRCCS, 00136 Rome, Italy; piergiorgiob@yahoo.it (P.B.); andreamazza83@hotmail.it (A.M.); dott.annalisapasquini@gmail.com (A.P.); serenadavino93@gmail.com (S.D.); gaia.deangelis03@icatt.it (G.D.A.); kiaracorigliano98@gmail.com (K.C.); alberta.marcolini@guest.policlinicogemelli.it (A.M.); massettimas@yahoo.it (M.M.); 2 School of Medicine and Surgery, Catholic University of the Sacred Heart, 00168 Rome, Italy; 3 Cardiovascular Research Institute, CARIM, 6629 ER Maastricht, The Netherlands; mdimauro1973@gmail.com (M.D.M.); marinosk@doctors.org.uk (M.K.); e.zancanaro96@gmail.com (E.Z.); p.meani@campus.unimib.it (P.M.); roberto.lorussobs@gmail.com (R.L.); 4 Department of Cardiovascular Surgery, Poliambulanza Foundation Hospital, 25124 Brescia, Italy; emmanuel.villa@gmail.com; 5 Department of Cardiac Surgery, Trent Cardiac Centre, Nottingham University Hospitals, Nottingham NG5 1PB, UK; 6 Department of Cardiac Surgery, San Raffaele Hospital, 20132 Milan, Italy; 7 Department of Cardiac Surgery and Transplantation, S. Camillo Hospital, 00152 Rome, Italy; guglielmo.saitto@gmail.com; 8 Heart and Vascular Centre, Maastricht University Medical Centre, 6229 HX Maastricht, The Netherlands

**Keywords:** aortic valve, bioprosthetic valve, sutureless, long-term outcomes

## Abstract

**Background:** In recent years, in case of aortic valve replacement (AVR), a significant increase in the use of bioprostheses has been observed. The Perceval sutureless bioprosthesis has proven to be safe and reliable in the short and mid-term, with limited but promising long-term results. An updated systematic review with the long-term results of patients who underwent a sutureless bioprosthesis implantation with a Perceval biological valve is herewith presented. **Methods:** Studies published between 2015 and 2024, including the long-term outcomes—with clinical as well as echocardiographic information for up to five years—of patients who underwent a Perceval implantation for AVR were selected from the published literature. The Cochrane GRADE system was used to assess the study quality, and the risk of bias in non-randomized studies (ROBINS-I) tool was used to evaluate studies. **Results:** Ten studies were selected with an overall number of 5221 patients. The long-term survival ranged from 64.8 to 87.9%, freedom from structural valve degeneration (SVD) from 96.1 to 100%, freedom from significant paravalvular leak from 98.5 to 100%, freedom from prosthetic endocarditis from 90.7 to 99%, and freedom from reintervention from 94 to 100%. The long-term mortality ranged from 6.5 to 27.4%. SVD was observed in 0–4.8% patients. Significant paravalvular leak was observed in 0–3.4% patients, and infective endocarditis was observed in 0–3.4%. A bioprosthesis-related reintervention at long-term follow-up was required for 0–4.3% of patients, and 1.7–7.1% of patients required a late new pacemaker implantation. The transprosthetic mean pressure gradient ranged from 9 to 14.7 mmHg, peak pressure gradient ranged from 17.8 to 26.5 mmHg, and EOA ranged from 1.5 to 1.7 cm^2^. **Conclusions:** This systematic review shows that there is still a paucity of data about sutureless bioprostheses. Nevertheless, the clinical results from prospective studies or retrospective series are encouraging. Medium- and long-term results seem to support the increasing use of this type of prosthesis.

## 1. Introduction

Aortic valve (AV) stenosis is the most common valvular heart disease. Despite the good results of transcatheter aortic valve implantation (TAVI) procedures [1,2], surgical aortic valve replacement (SAVR) is still preferred in many operable patients with severe AV disease. In patients eligible for SAVR, bioprostheses are increasingly preferred over mechanical prostheses. Indeed, in addition to the aging of patients, the introduction of surgical bioprostheses with expected improved hemodynamics and durability, along with the favorable results obtained with transcatheter valve-in-valve (V-in-V) procedures in case of bioprosthetic degeneration, encouraged the expanded use of bioprostheses in a wide range of patients [3,4,5]. Furthermore, the higher number of elderly patients with several comorbidities also increased the surgical risk for perioperative morbidity and mortality [6,7].

The need to perform less invasive interventions with shorter cardiopulmonary bypass (CPB) and aortic cross-clamping (ACC) times, thus minimizing the surgical and perioperative risks, favored the introduction of sutureless and rapid-deployment valves [8,9,10,11,12,13,14,15]. These bioprosthetic valves are characterized by a simplified and reproducible implant procedure and shorter surgical times, and, different from TAVI, the native aortic cusps are removed and an accurate decalcification of the annulus can be achieved.

The Perceval bioprosthesis (Corcym UK Limited, London, UK) has encountered wide favor among cardiac surgeons for its easy implantability, safety, excellent hemodynamic performance, and satisfactory postoperative results [8,9,10,11,12,13,14,15,16]. Mid-term and long-term data are, however, still limited, but the first meta-analyses have shown promising results [15,16].

Here, we present a systematic review of studies published over the past 15 years to provide updated information on the reliability and durability of Perceval bioprostheses in the long term.

## 2. Methods

This systematic review was performed according to the Preferred Reporting Items for Systematic Review and meta-analysis statement (PRISMA) Guidelines [17].

Results were reported following PRISMA Guidelines [17], ensuring transparency and reproducibility. This systematic review was not formally registered; however, all procedures in this study were conducted in strict adherence to the PRISMA Guidelines to ensure methodological rigor, as recently reported [18].

Since this is just a systematic review, no pooling analyses or other statistical analyses were performed.

As this is an updated review of already published studies, the preparation of a protocol was not considered necessary. The included studies are publicly available, and all data used in this analysis are reported in the text.

### 2.1. Eligibility Criteria

Studies included in this review were original articles only, prospective or retrospective, published in the English language, and dealing with adult patients (>18 years old) who underwent a Perceval sutureless aortic bioprosthesis implantation, either for isolated AVR or concomitant procedures. Only the studies presenting long-term data and reporting results for up to five years were considered.

The studies of other surgical bioprostheses, of children’s cases (patients <18 years old) or non-human subjects, with a maximum follow-up of less than 5 years, and articles other than original data (case reports, editorials, commentaries, conference abstracts, or other unpublished data) were excluded.

In case of multiple publication, with the same group of patients included in more than one study (e.g., single-center studies published before multicenter studies) [19,20], only the most recent research was considered, with the largest number of patients and the longest follow-up.

### 2.2. Information Sources

Studies were extracted from five databases (PubMed, SCOPUS, EMBASE, MEDLINE, and the Cochrane Database). Furthermore, references from included publications and from the main cardiac surgery specialty journals (*Annals of Thoracic Surgery*, *Journal of Thoracic and Cardiovascular Surgery*, *European Journal of Cardiothoracic Surgery*, and *Interactive/Interdisciplinary Journal of Cardiothoracic Surgery*) over the past 15 years were also screened. The search was last performed on 20 October 2024.

#### 2.2.1. Search Strategy

**Figure 1:**
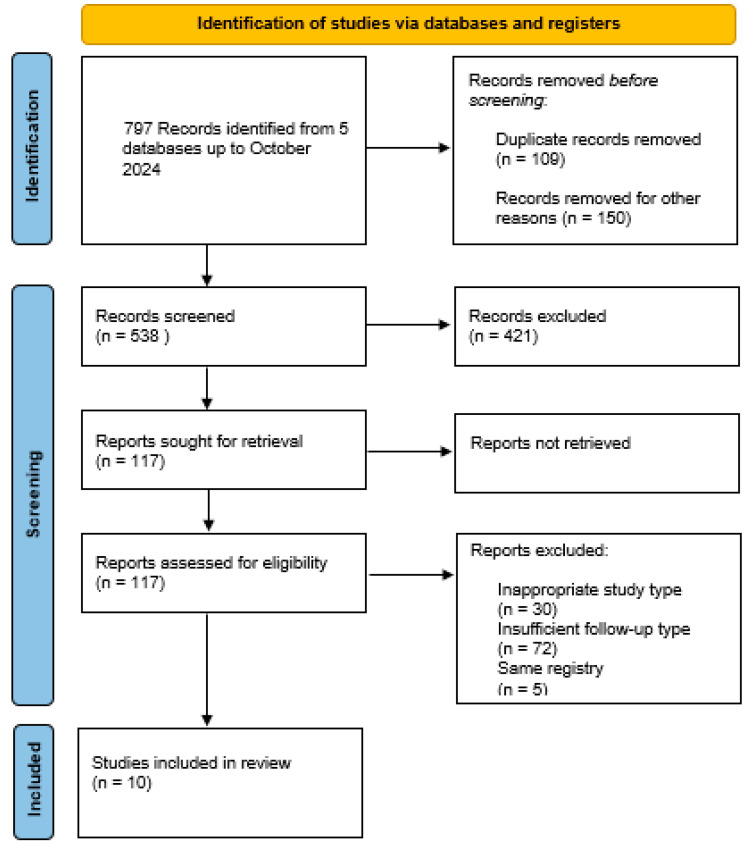
A PRISMA diagram for the identification of studies by way of databases and registers.

Figure 1 summarizes the details of the search strategy.

The search strategy was characterized by both MeSH headings and keywords.

The keywords selected for the study search were “sutureless”, “Perceval”, “aortic valve bioprostheses”, “long-term”, and “long-term durability”. The references from the retrieved studies initially selected were included if they met the inclusion criteria. All retrieved articles were reviewed by two independent reviewers (GAC and EV). Disagreements were resolved by discussion with a third senior investigator (RL). In case of missing data in single studies, corresponding authors were privately contacted. The search strategy was discussed with all authors and verified by an independent librarian at the Cardiovascular Research Institute of Maastricht. The Cochrane GRADE system was used to assess the study quality and the risk of bias in non-randomized studies (ROBINS-I) tool [19], and it was used to evaluate the studies.

#### 2.2.2. Data Collection Process

Three independent reviewers (GAC, EV, and MdM) collected data from each report. The data were extracted to Microsoft Excel by independent reviewers that subsequently checked each other’s entries and data integrity.

#### 2.2.3. Data Abstraction/Synthesis

Data items collected included the following: study period, number of patients, follow-up time, mean age, sex, body surface area (BSA), prevalence of diabetes, chronic obstructive pulmonary disease (COPD), dyslipidemia, renal failure, previous neurovascular events, peripheral vascular disease, New York Heart Association (NYHA) functional class, Logistic Euroscore, Society of Thoracic Surgeons (STS) SCORE, functional pathology of aortic valve (AV) disease, preferred surgical technique, bicuspid aortic valve (BAV) patients, reintervention and combined procedures, cardiopulmonary bypass (CPB) time, and aortic cross-clamping (ACC) time.

### 2.3. Outcomes

The primary outcomes of this systematic review were long-term survival and long-term bioprosthesis durability in terms of freedom from structural valve degeneration (SVD), from significant paravalvular leak (PVL), from infective prosthetic valve endocarditis (PVE), and from AV reintervention. Secondary outcomes included mortality; cardiovascular mortality; incidence of SVD, PVL, and PVE; reintervention rate; reintervention for SVD (surgical bioprosthesis explant or TAVI valve in valve); prosthesis explant for PVL and PVE; incidence of stroke; incidence of pacemaker implantation; and hemodynamic performance (mean and peak transprosthetic pressure gradient, MPG and PPG; effective orifice area, EOA). Only the long-term results were considered. Consequently, 30-day data and patients lost at follow-up were excluded from the final analysis.

### 2.4. Ethical Approval

No ethical approval was sought as this was a systematic review.

## 3. Results

Studies were identified and selected according to the PRISMA flow-chart (Figure 1). After extensive research, ten studies, published between 2015 and 2024, meeting the aforementioned inclusion criteria, were identified, including 5221 patients who underwent AVR with a sutureless valve implantation [20,21,22,23,24,25,26,27,28,29]. Three studies were prospective, [20,21,26], and seven were retrospective [22,23,24,25,27,28,29]. Nine studies reported the results of patients who underwent a Perceval implantation only. In one study [23], from an initial sample of 967 patients, after propensity score matching, two matched groups were obtained, comparing the patients who underwent a Perceval implantation and the patients who underwent TAVI (Table 1).

### 3.1. Patients

Patient characteristics are summarized in Table 2. Mean (SD, standard deviation) age ranged from 71.2 (7.6) to 80.4 (3.8) years, and the majority of patients were female, except in the series of patients presented by White et al. [23], and Dokollari et al., where males made up 63 and 54.4%, respectively. Diabetes prevalence ranged from 3.8 to 40.6%. Prevalence of dyslipidemia was reported in four studies only, ranging from 56 to 75.2%. Among preoperative comorbidities, COPD ranged from 13 to 18.5%, renal failure from 3.6 to 14.8% and PVD from 4.1 to 26%. The STS score was reported in only three studies, ranging from 5.8 to 7.2%.

The operative data are reported in Table 3. Most patients were operated on for isolated severe AV stenosis (65.3–100%), and the most frequently performed surgical approach was full median sternotomy (53.2–100%). A minimally invasive approach (mini-sternotomy or right mini-thoracotomy) was accomplished in 29.9–70.3% of patients.

### 3.2. Primary Outcomes

Long-term follow-up survival ranged from 64.8 to 87.9%, freedom from SVD ranged from 96.1 to 100%, freedom from paravalvular leak ranged from 98.5 to 100%, freedom from endocarditis ranged from 90.7 to 99%, and freedom from reintervention ranged from 94 to 100%.

### 3.3. Secondary Outcomes

Secondary outcomes are reported in Table 4. Overall long-term mortality ranged from 6.5 to 27.4%, and death for cardiovascular causes ranged from 1.7 to 18%.

An AV reintervention at long-term follow-up was required for 0–4.3% of patients.

The incidence of significant SVD ranged between 0 and 4.8%, and the rate of reintervention for SVD ranged from 0 to 4%; 0–1.2% of patients underwent surgical bioprosthesis explant, and 0–3.1% underwent transcatheter V-in-V. Significant PVL was observed in 0–3.4% patients with a very exiguous rate of reintervention of 0–0.5%. Infective PVE was observed in 0–3.4% of patients, and 0–1.4% required reintervention.

A late neurovascular event (stroke) was observed in 0–10% of patients, and 1.7–7.1% of patients required a late new pacemaker implantation.

At the long-term follow-up echocardiogram, mean transprosthetic MPG ranged from 9 to 14.7 mmHg, PPG ranged from 17.8 to 26.5 mmHg, and EOA ranged from 1.5 to 1.7 cm^2^.

## 4. Discussion

In recent decades, an increasing use of biological artificial valves for AVR has been observed [3,5]. The reasons for this preference may be identified in the patients’ desire to avoid life-long anticoagulant therapy, in the aging population, and in the satisfactory results obtained with the V-in-V procedures in the case of prosthesis valve degeneration, therefore not requiring surgical reintervention. The aging population, with patients presenting multiple comorbidities and therefore with a higher risk of perioperative complications, prompted a search for bioprostheses implantable with simplified techniques and requiring shorter CPB and ACC times, to reduce such an operative risk [14,15]. The Perceval bioprosthesis proved to be effective, safe, and reliable, needing a simplified and reproducible implantation technique and having an optimal hemodynamic performance, as previously reported [8,9,10,11,12,13,14,27,28,29,30]. The long-term clinical and hemodynamic results of this type of bioprosthesis are still limited, but in recent years, several studies have reported the first long-term results.

William et al. [15] conducted the first meta-analysis with mid-term results of patients who underwent either a Perceval or rapid-deployment INTUITY (Edwards Lifesciences, Irvine, CA, USA) valve implantation. The results were promising in terms of survival, freedom from complications, durability, and hemodynamic performance.

The increasing number of studies reporting the late results of sutureless prostheses led to the first meta-analysis by Jeoliffe et al. [16], with accurate mid-term clinical and hemodynamic results. In 2022–2024, new large studies reported innovative data on the long-term performance and reliability of the Perceval bioprosthesis.

In 2015, Meuris B et al. first reported late results (median follow-up of 4.2 years) of the first series of 30 patients who underwent a Perceval implantation, after having described promising 30-day results. Mid-term results in terms of survival and durability were optimal, with no cases of reintervention or SVD and only one case of non-structural valve deterioration. Subsequent studies published between 2019 and 2022 confirmed the excellent durability of the Perceval bioprosthesis, with good performance also in the long term. In 2023, Jolliffe et al. published the first meta-analysis focused on the mid-term results of Perceval bioprostheses [16]. At a mean follow-up of 4.1 years, the weighted pooled estimate for overall mortality was 11.2%, and the five-year survival was 79.5%. The authors reported an incidence of SVD of 1.5% (0.7–2.6), and 3.6% of patients (2.2–5.4) presented paravalvular leak. A reintervention was required for 2.3% (1.3–3.4) of patients, and among them 0.4% patients required surgical explant or a valve-in-valve procedure for SVD.

In this review, data obtained from 10 studies showed a survival rate ranging from 64.8 to 87.9 and a mortality rate of 4.4–27.4%. The results were even more satisfactory in terms of durability and freedom from reintervention. Indeed, freedom from reintervention was 94–100%, and a reintervention was required for 0–4.3% of patients. Similarly to Jolliffe et al., SVD was observed in 0–4.8%, with an even lower reintervention rate for SVD (surgical explant or TAVI valve in valve) observed in 0–4% of patients. Infective PVE was observed in 0–3.4% patients, and 0–1.4% required reintervention.

The risk of significant PVL appeared acceptable and very low compared to TAVI procedures [31,32]. The surgical removal of aortic cusps and accurate annular decalcification, thus favoring the optimal adherence of the stent frame to the aortic annulus, seemed to represent significant advantages in preventing early and late PVL. This review also confirmed the data reported in 2016 by Shrestha M et al. [30], having presented the cumulative results of 731 patients from three prospective multicentric trials.

The Perceval bioprosthesis appeared to be reliable in many scenarios. The easy and reproducible implantability, collapsed configuration prior to deployment, and reduced tissue manipulation provided significant technical advantages, mainly in patients with small aortic annuli, in reinterventions, in minimally invasive surgery, or in patients with challenging anatomy, such as obese patients [14,33,34,35,36].

Some concerns were expressed for patients with bicuspid aortic valve (BAV). The implantation of sutureless valves in patients with BAV remains indeed controversial, and the implantation of sutureless prostheses in BAV has been discouraged in previous years, since the irregular elliptic shape of the aortic bicuspid annulus, asymmetric height of commissures, and unequal Valsalva sinuses may be responsible for the poor stability of the sutureless bioprostheses with possible dislocation and paravalvular leak. Nevertheless, in 2015, Nguyen A et al. [37], reported a series of 25 patients with type I BAV who underwent a Perceval implantation. No cases of PVL, valve displacement, prosthesis migration, or SVD were observed at 1 year. However, in 2018, the first case of a Perceval bioprosthesis displacement was observed in a type I BAV annulus at 22 months from the AVR, with both intravalvular and paravalvular leaks [38]. At reoperation and Perceval explantation, no signs of SVD were observed. More recently, some authors [39,40] have recommended the use of sutureless prostheses in BAV type I and in selected cases of BAV type II when the two commissures present the same height. Conversely, BAV type 0 is considered an absolute contraindication. Therefore, sutureless prostheses in BAV could be chosen in selected cases, considering the symmetry of the aortic annulus and the equality of the heights of the aortic commissures. In this review, not all selected studies reported specific results on BAV patients. In the study by Muneretto et al. [23], nine (1.9%) patients underwent reoperation because of valve malpositioning with significant paravalvular leak. Among them, four patients with elliptic annulus presented a Sievers type I BAV. Conversely, Szecel et al. [22], reported the implantation of a Perceval bioprosthesis in 11 patients with type I BAV, and no cases of late valve malpositioning and significant paravalvular leak were observed.

The incidence of late stroke appears surprisingly significant, ranging between 0 and 10%. These data should be taken with great caution, as only one of the considered studies [27], with a limited number of patients, reports such an incidence, likely due to the population risk profile, which is different from the other larger studies that report an incidence of stroke ranging between 0 and 4.1% [20,21,22,23,24,25,26,28].

In this review, Perceval bioprosthesis was also shown to be safe and reliable in the long term, with optimal durability, freedom from reintervention for SVD, and a low rate of PVL, further improved compared to a previous meta-analysis and comparable to stented sutured bioprostheses [41,42,43]. In the studies selected for this systematic review, in case of SVD, transcatheter V-in-V procedures were performed with satisfactory results. The feasibility of V-in-V TAVI in the Perceval bioprosthesis has been previously reported, with no cases of patient–prosthesis mismatch or coronary obstruction [44].

Despite the relatively limited number of heterogeneous studies that met the inclusion criteria for this review, the results of this analysis confirmed the excellent hemodynamics. The postoperative gradients and EOA were optimal, even better compared to stented bioprostheses. The low-profile Nitinol frame and the absence of the sewing ring entail a larger EOA with a reduced transprosthetic pressure gradient. Furthermore, due to the recent improvement in the operating techniques [45,46], aimed at reducing oversizing, the rate of pacemakers, and the transvalvular pressure gradients, a possible decrease in the risk of SVD would be expected.

## 5. Conclusions

This systematic review shows that there are still too little data on sutureless bioprostheses in the literature. Nevertheless, the medium- and long-term results are promising in terms of late reliability and durability and seem to support the increasing use of this type of prosthesis. Future randomized studies and comparative data, with larger series of patients, should provide more definitive results.

## Figures and Tables

**Table 1:** Selected studies.

Author	Study Design	Year	Trial/Registries	Study Period	Patient Number	Median Follow-Up (Years)	Maximum Follow-Up (Years)	Risk of Bias (ROBINS-1)
Meuris et al. [20]	PC	2015	Pilot (5 years)	2007–2008	30	4.2	5	Serious
Fishlein et al. [21]	PC	2021	CAVALIER	2010–2013	658	3.8	5	Serious
Szecel et al. [22]	RC	2021	Institutional data	2007–2017	468	3.1	11.2	Serious
Muneretto et al. [23]	RC	2022	Institutional data	2008–2015	481	5	-	Moderate
White et al. [24]	RC	2022	Institutional data	2013–2019	295	2.4	5	Serious
Pollari et al. [25]	RC	2023	Institutional data	2010–2020	547	3.8	10.6	Serious
Concistre et al. [26]	PC	2023	SURE-AVR	2011–2021	1652	1.2	8	Serious
Dokollari et al. [27]	RC	2023	Institutional data	2013–2020	101	1.5	7	Serious
Lamberigts et al. [28]	RC	2024	Institutional data	2007–2019	784	7	13.6	Serious
Shizas N et al. [29]	RC	2024	Institutional data	2013–2020	205	6.7	10	Moderate

ROBINS-1: risk of bias in non-randomized studies tool; PC: prospective cohort; RC: retrospective cohort.

**Table 2:** Patient characteristics.

Author	Age Mean (SD) Years	Gender (Male) *n* (%)	BSA Mean (SD) m^2^	Diabetes *n* (%)	COPD *n* (%)	Dyslipidemia *n* (%)	Renal Failure *n* (%)	Previous NV Events *n* (%)	PVD *n* (%)	NYHA > III *n* (%)	Euroscore Mean (SD)%	STS Score Mean (SD)%
Meuris et al. [20]	80.4 (3.8)	8 (27)	1.8 (0.2)	N/A	N/A	N/A	N/A	N/A	N/A	30 (100)	13.1 (7.2)	N/A
Fishlein et al. [21]	78.3 (5.6)	234 (35.6)	1.8 (0.2)	191 (29)	103 (15.7)	N/A	97 (14.8)	75 (11.4)	112 (17)	418 (63.5)	10.2 (7.8)	7.2 (7.4)
Szecel et al. [22]	79 (5)	206 (44)	1.8 (0.2)	116 (25)	75 (16)	N/A	N/A	N/A	122 (26)	278 (59.4)	N/A	5.8 (5.5)
Muneretto et al. [23]	79 (5)	174 (36.2)	N/A	154 (32)	89 (18.5)	N/A	59 (12.3)	31 (6.4)	81 (16.8)	285 (59.3)	13.6 (18.4)	5.7 (6.4)
White et al. [24]	72.4 (9.9)	188 (63.7)	N/A	75 (25.4)	40 (13.6)	166 (56.3)	12 (4.1)	14 (4.7)	12 (4.1)	N/A	N/A	N/A
Pollari et al. [25]	76.4 (5.2)	268 (49)	1.88 (0.2)	170 (31)	73 (13)	409 (75)	20 (3.6)	15 (3)	161 (29.4)	N/A	13 (11)	N/A
Concistre et al. [26]	75.3 (7)	761 (46)	N/A	514 (31.1)	228 (13.8)	937 (56.7)	184 (11.1)	107 (6.5)	103 (8.5)	N/A	N/A	N/A
Dokollari et al. [27]	71.2 (7.6)	55 (54.4)	1.9 (0.25)	41 (40.6)	N/A	76 (75.2)	N/A	14 (14)	N/A	61 (61)	3.5 (4.4)	N/A
Lamberigts et al. [28]	78.5 (5.8)	279 (48.3)	1.8 (0.2)	30 (3.8)	119 (15.2)	N/A	N/A	N/A	196 (25)	406 (51.7)	N/A	N/A
Shizas N et al. [29]	76.4 (34.1)	70 (34.1)	N/A	N/A	N/A	N/A	N/A	N/A	N/A	N/A	N/A	N/A

BSA: body surface area; COPD: chronic obstructive pulmonary disease; NV: neurovascular; PVD: peripheral vascular disease; NYHA: New York Heart Association functional class; STS: Society of Thoracic Surgeons; N/A: not applicable or not available data.

**Table 3:** Operative data.

Author	FS *n* (%)	Minimally Invasive *n* (%)	Redo Operation *n* (%)	Combined Operation *n* (%)	Combined CABG *n* (%)	CPB Time Mean (SD) Min	ACC Time Mean (SD) Min	AV Sten *n* (%)	AV Reg *n* (%)	AV Sten and Reg *n* (%)	BAV *n* (%)
Meuris et al. [20]	30 (100)	N/A	3 (10)	14 (46.6)	14 (46.6)	46.4 (6.7)	29.3 (8)	23 (76.7)	0 (0)	7 (23.3)	N/A
Fishlein et al. [21]	439 (66.7)	219 (33.3)	446 (68) *	207 (31.5)	154 (23.4)	58.7 (20.2)	35.5 (12.4)	430 (65.3)	2 (0.3)	226 (34.3)	12 (1.8)
Szecel et al. [22]	328 (70)	140 (29.9)	N/A	267 (57)	184 (39)	66 (22)	39 (13)	N/A	8 (1.7)	N/A	11 (2.3)
Muneretto et al. [23]	256 (53.2)	225 (46.7)	35 (7.3)	N/A	N/A	56 (25)	35 (16)	481 (100)	0 (0)	0 (0)	31 (6.4)
White et al. [24]	N/A	N/A	N/A	94 (31.8)	N/A	N/A	N/A	N/A	N/A	N/A	N/A
Pollari et al. [25]	162 (29.6)	385 (70.3)	21 (4)	173 (31.6)	141 (26)	59.4 (20)	36.1 (11)	544 (99.1)	3 (0.5)	0 (0)	69 (13)
Concistre et al. [26]	899 (54.4)	744 (45)	270 (16.3)	593 (35.9)	426 (25.8)	77.4 (30.8)	51 (20.5)	1233 (74.6)	89 (5.4)	300 (18.2)	132 (8)
Dokollari et al. [27]	101 (100)	0 (0)	24 (24)	0 (0)	0 (0)	65 (29.6)	47.3 (21.3)	88 (88)	0 (0)	13 (13)	25 (25)
Lamberigts et al. [28]	541 (69)	243 (31)	N/A	435 (55.4)	239 (30.5)	N/A	N/A	N/A	N/A	N/A	N/A
Shizas N et al. [29]	96 (47)	109 (53)	N/A	68 (33)	63 (30.7)	59.1 (15.3)	49.1 (13.4)	185 (90.2)	4 (1.9)	N/A	N/A

FS: full sternotomy; CPB time: cardiopulmonary bypass time; ACC time: aortic cross-clamping time; AV: aortic valve; Sten: stenosis; Reg: regurgitation; BAV: bicuspid aortic valve; *: pacemaker implantations were included; N/A: not applicable or not available data.

**Table 4:** Follow-up results.

Author	N	Deaths *n* (%)	cv Deaths *n* (%)	SVD ** n (%)	PVL ** n (%)	PVE *n* (%)	Overall Reintervention *n* (%)	Redo for SVD *** n (%)	Surgica Redo (Explant) for SVD *n* (%)	V-in-V for SVD *n* (%)	Redo (Explant) for PVL *n* (%)	Redo (Explant) for PVE *n* (%)	NV Events *n* (%)	PMK *n* (%)	Valve Thrombosis *n* (%)	Hemolysis *n* (%)
Meuris et al. [20]	29	6 (20.6)	1 (3.4)	0 (0)	1 (3.4)	2 (7)	0 (0)	0 (0)	0 (0)	0 (0)	0 (0)	0 (0)	0 (0)	1 (3.4)	0 (0)	0 (0)
Fishlein et al. [21]	599	131 (21.9)	59 (9.8)	13 (2.1)	6 (1)	17 (2.8)	24 (4)	13 (2.1)	7 (1.2)	6 (1)	0 (0)	8 (1.3)	18 (3)	15 (2.5)	0 (0)	1 (0.2)
Szecel et al. [22]	453	97 (21.4)	27 (5.9)	10 (2.2)	14 (3)	5 (1.1)	5 (1.1)	0 (0)	0 (0)	0 (0)	0 (0)	5 (1.1)	N/A	11 (2.4)	0 (0)	N/A
Muneretto et al. [23]	287 *	46 (16.1)	5 (1.7)	2 (0.7)	1 (0.3)	2 (0.7)	3 (1)	1 (0.3)	1 (0.3)	0 (0)	1 (0.3)	2 (0.7)	4 (1.4)	5 (1.8)	0 (0)	N/A
White et al. [24]	288	19 (6.5)	N/A	N/A	N/A	N/A	0 (0)	N/A	N/A	N/A	N/A	N/A	12 (4.1)	20 (7)	0 (0)	N/A
Pollari et al. [25]	529	110 (20.7)	13 (2.4)	23 (4.8)	1 (0.2)	10 (1.9)	23 (4.3)	19 (4)	4 (0.8)	15 (3.1)	0 (0)	4 (0.7)	5 (0.9)	9 (1.7)	0 (0)	N/A
Concistre et al. [26]	1639	127 (7.7)	55 (3.3)	10 (0.6)	0 (0)	14 (0.8)	23 (1.4)	10 (0.6)	1 (0.06)	9 (0.5)	6 (0.3)	7 (0.4)	12 (0.7)	48 (3)	0 (0)	N/A
Dokollari et al. [27]	99	12 (12)	5 (5)	0 (0)	0 (0)	1 (1)	0 (0)	0 (0)	0 (0)	0 (0)	0 (0)	0 (0)	10 (10)	5 (5)	N/A	N/A
Lamberigts et al. [28]	758	208 (27.4)	N/A	15 (1.9)	9 (1.2)	13 (1.7)	14 (1.8)	3 (0.4)	0 (0)	3 (0.4)	0 (0)	11 (1.4)	11 (1.4)	N/A	N/A	N/A
Shizas N et al. [29]	196	47 (24)	35 (18)	1 (0.5)	1 (0.5)	0 (0)	5 (2.5)	1 (0.5)	0 (0)	1 (0.5)	1 (0.5)	0 (0)	N/A	14 (7.1)	N/A	N/A

*: matched; **: equal or more than moderate; ***: surgical redo + TAVI valve in valve; CV: cardiovascular; SVD: structural valve degeneration; PVL: paravalvular leak; PVE: prosthetic valve endocarditis; V-in-V: valve in valve; NV: neurovascular; PMK: pacemaker; N/A: not applicable or not available data.
